# Quantification of Staphylococcal Enterotoxin A Variants at Low Level in Dairy Products by High-Resolution Top-Down Mass Spectrometry

**DOI:** 10.3390/toxins16120535

**Published:** 2024-12-11

**Authors:** Nina Aveilla, Cécile Feraudet-Tarisse, Dominique Marcé, Abdelhak Fatihi, François Fenaille, Jacques-Antoine Hennekinne, Stéphanie Simon, Yacine Nia, François Becher

**Affiliations:** 1 CEA, INRAE, Département Médicaments et Technologies pour la Santé (DMTS), SPI, Université Paris-Saclay, 91191 Gif-sur-Yvette, France; nina.aveilla@cea.fr (N.A.); cecile.feraudet-tarisse@cea.fr (C.F.-T.); dominique.marce@cea.fr (D.M.); francois.fenaille@cea.fr (F.F.); stephanie.simon@cea.fr (S.S.); 2 Laboratory for Food Safety, French Agency for Food, Environmental and Occupational Health & Safety (ANSES), Université Paris-Est, 94700 Maisons-Alfort, France; abdelhak.fatihi@anses.fr (A.F.); jacques-antoine.hennekinne@anses.fr (J.-A.H.); yacine.nia@anses.fr (Y.N.)

**Keywords:** mass spectrometry, variants, sensitivity, staphylococcal enterotoxins, intact protein

## Abstract

Food poisoning outbreaks frequently involve staphylococcal enterotoxins (SEs). SEs include 33 distinct types and multiple sequence variants per SE type. Various mass spectrometry methods have been reported for the detection of SEs using a conventional bottom-up approach. However, the bottom-up approach cannot differentiate between all sequence variants due to partial sequence coverage, and it requires a long trypsin digestion time. While the alternative top-down approach can theoretically identify any sequence modifications, it generally provides lower sensitivity. In this study, we optimized top-down mass spectrometry conditions and incorporated a fully ^15^N-labeled SEA spiked early in the protocol to achieve sensitivity and repeatability comparable to bottom-up approaches. After robust immunoaffinity purification of the SEA, mass spectrometry signals were acquired on a Q-Orbitrap instrument operated in full-scan mode and targeted acquisition by parallel reaction monitoring (PRM), enabling the identification of sequence variants and precise quantification of SEA. The protocol was evaluated in liquid and solid dairy products and demonstrated detection limits of 0.5 ng/mL or ng/g in PRM and 1 ng/mL or ng/g in full-scan mode for milk and Roquefort cheese. The top-down method was successfully applied to various dairy products, allowing discrimination of contaminated *versus* non-contaminated food, quantification of SEA level and identification of the variant involved.

## 1. Introduction

Staphyloccocal enterotoxins (SEs) are responsible for the occurrence of many cases of food poisoning outbreaks (FPOs) each year. They represent the second leading cause of FPOs involving bacterial toxins in Europe, as reported in the 2022 European Food Safety Authority (EFSA) report [1]. Consumption of food contaminated with staphylococcal enterotoxins causes nausea, vomiting, abdominal cramping and diarrhea, typically appearing very quickly within 30 min to 8 h after ingestion [2]. Numerous FPOs caused by SEs have been reported in different types of dairy food products, such as ice cream [3], pasteurized milk products [4] and cheeses [5,6,7]. Due to their richness in various nutrients and neutral pH, dairy products are prone to contamination by *Staphylococcus aureus*, which can readily grow in these favorable conditions and produce enterotoxins [8]. To ensure food chain security, it is essential to have specific, sensitive, robust and rapid analytical methods for the early identification of contaminated dairy food products.

A wide variety of SEs have been identified, encompassing up to 33 different types [9], ranging in size from 24 to 30 kDa. Each SE type exhibits sequence variants with differences of 1 to 50 amino acids. Merda et al. [10] proposed an 84% sequence identity threshold, above which SEs are considered different variants. The high sequence homology between variants presents significant challenges for conventional SE detection methods. Immunoassays such as ELISAs [11,12] or LFA [12,13] can identify SE types but are unable to distinguish between all potential variants. In bottom-up mass spectrometry [14,15], the process of digestion into peptides combined with the inherently partial sequence coverage prevents the detection of combinatorial sequence modifications of the variants. Furthermore, the sequence modifications can alter the signature peptides monitored, potentially leaving variants undetected and resulting in false-negative results [16].

Top-down mass spectrometry offers the capability to detect, discriminate and quantify SE variants when combining intact protein mass measurement by high-resolution full-scan acquisition mode and targeted analysis of the most abundant charge states by using the parallel reaction monitoring (PRM) mode, as demonstrated for SEA by Lefebvre et al. (2021) [17]. Top-down mass spectrometry also has the advantage of being faster, as it analyzes the intact protein without prior digestion into peptides. However, top-down was not applied to food samples because the detection limit exceeded the levels typically found in SFPO episodes [18,19,20]. Consequently, the method could mainly confirm which variant was produced by a strain under optimal culture conditions. 

SEA is the most common toxin involved in SFPOs [19,20,21] and was, therefore, the focus of this work. We report a more sensitive top-down method capable of rapidly quantifying SEA and distinguishing between variants. Optimization of the sample preparation protocol, including enhanced immunoprecipitation conditions and the early addition of a labeled toxin, led to more efficient and robust SEA purification, as well as reliable quantification in protein-rich dairy matrices. In addition, mass spectrometry acquisition parameters were adapted to better compensate for the signal dispersion inherent in top-down proteomics [22]. The enhanced top-down method was successfully applied to various dairy products, including milk, cheeses and ice cream, with a detection limit below 1 ng/mL, similar to bottom-up approaches, showing its applicability to real cases of FPO.

## 2. Results

We recently introduced the first top-down method for detecting SEA through immunopurification and intact protein analysis by high-resolution mass spectrometry (HRMS) [17]. Interestingly, the method was able to rapidly detect SEA in buffer or culture media and constituted the only proteomic method capable of distinguishing the four main variants of the toxin. However, the sensitivity was five times lower compared to bottom-up methods [15], and the method was not applied to real food extracts representative of samples collected during FPOs. Also, internal standardization should ideally be based on the addition of a stable isotope-labeled analog of the toxin [23,24].

In this work, we enhanced our initial top-down assay for SEA by implementing several key optimizations throughout the protocol. These included the production and the early addition in the protocol of a stable isotope ^15^N labeled SEA toxin, as well as the refinement of the immunoprecipitation and mass spectrometry acquisition conditions. The final protocol was evaluated on real dairy samples, including milk, diverse French cheeses, and ice cream, achieving a five-fold improvement in sensitivity over the initial protocol.

### 2.1. Production and Characterization of ^15^N Isotope-Labeled SEA_3_ for Protocol Normalization

**Figure 1:**
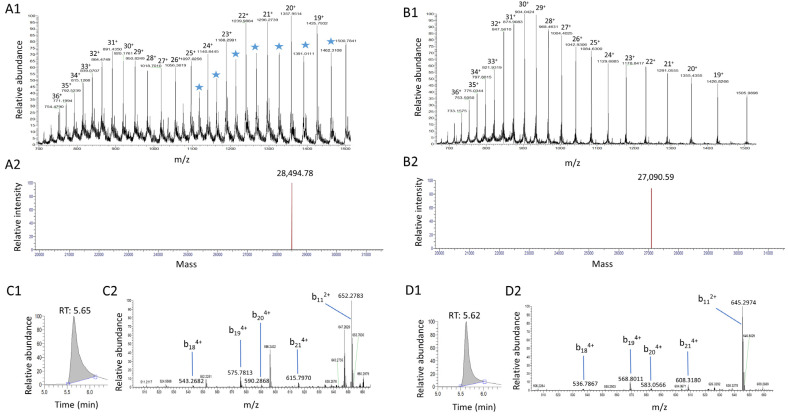
Intact purified ^15^N SEA_3_ (**A**) and SEA_3_ (**B**) at 10 µg/mL analyzed by HRMS/MS. Intact ^15^N SEA_3_ (**A1**) and SEA_3_ (**B1**) analyzed by full-scan HRMS and corresponding deconvoluted spectra (**A2**,**B2**). Spectra were deconvoluted with the software «Protein Deconvolution» version 4.0 from Thermo Scientific. The observed mass matched with a theoretical mass of 28,494.70 Da corresponding to 341 labeled nitrogens, a loss of one histidine in the His-Tag, and the expected disulfide bridge between the two cysteines. Blue star: additional peaks attributed to a dimeric form. HRMS/MS spectra of intact ^15^N SEA_3_ (**C2**) and SEA_3_ (**D2**) using HCD and observed retention times (**C1**,**D1**).

Normalization of the analytical protocol is essential for achieving robust and accurate quantification by mass spectrometry. The addition of a labeled version of the protein early in the protocol helps compensate for variation in recovery during sample preparation and in mass spectrometry ionization [25,26]. In this study, we produced a labeled version of the consensus sequence variant P0A0L2, named SEA_3_, using the corresponding plasmid containing the gene for protein expression [12]. The bacterial culture was carried out in a minimal medium where the only nitrogen source was ^15^N-labeled ammonium chloride [27]. Several bacterial conditions of incubation (temperature, concentration of isopropyl β-D-1-thiogalactopyranoside (IPTG), time of incubation) were evaluated to increase the production yield of ^15^N-labeled SEA (Figure S1). Final incubation conditions were set at 30 °C with 1000 µM IPTG for 22 h. The labeled protein was characterized by gel electrophoresis and bottom-up and top-down mass spectrometry. Gel electrophoresis indicated that the protein in fractions 3–4 had the expected size and the best purity (Figure S2). Mass spectrometry characterization was performed first by bottom-up proteomics after trypsin digestion. The mass shift of the three most intense peptides of SEA was in agreement with fully labeled sequences (Figure S3). The intact native SEA_3_ variant and labeled ^15^N SEA_3_ were then analyzed by HRMS to further confirm the sequence and the isotope incorporation (Figure 1A,B). The additional peaks between *m*/*z* 1000 and 1500 (Figure 1A) were attributed to a minor dimeric form of ^15^N SEA_3_, considering that the two cysteine residues contained in the amino-acid sequence may form disulfide bonds during protein expression in *E. coli* [28]. No other protein was detected in the mass spectra, illustrating the purity of the labeled SEA. Comparison of the charge state distributions of native SEA_3_ and ^15^N SEA_3_ suggests the presence of two unfolded forms of ^15^N SEA_3_, likely due to the remaining His-Tag (Figure 1(A1,B1)). The deconvoluted mass spectra reported a single peak at 28,494.78 Da, as shown in Figure 1(A2), which matched with the theoretical mass of the sequence of a fully labeled ^15^N SEA_3_ at 28,494.70 Da (+0.08 Da, +2.8 ppm) and the presence of the expected bridge between the two cysteines, similarly to the native SEA_3_ (Figure 1(B2)). After MS/MS co-selection and fragmentation of the nine main charge states of ^15^N SEA (i.e., z = 28 to 36) (Figure 1(C2,D2)), identical fragment ions of the native toxin were detected (Table S1). The shift in mass of the fragment ions (Table S1) was in agreement with a fully labeled sequence with no unlabeled fragment ions detected. We also observed additional fragment ions in the ^15^N SEA spectra, which likely resulted from the fragmentation of the dimeric form. Globally, intact and bottom-up results confirmed the purity, the primary sequence and total isotope labeling of the recombinant labeled protein, also showing the absence of any potential interferences on the signal of unlabeled SEA_3_. The identical retention time (+0.03 min) observed for intact ^15^N SEA_3_ and native SEA_3_ (Figure 1(C1,D1)) with the overlap of the elution peaks suggests that the labeled SEA should cover problems with ion suppression and matrix effects. 

### 2.2. Optimization of the Top-Down Analytical Protocol for Maximum Sensitivity 

We modified the sample preparation for higher recovery of the toxin in complex food matrices, while mass spectrometry acquisition parameters were also optimized for better detection performances. 

**Figure 2:**
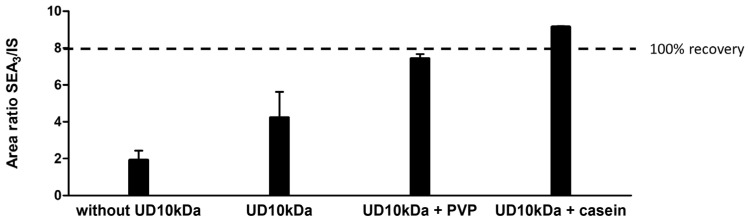
Measurement of the SEA_3_ to ^15^N SEA_3_ ratio using different filtration conditions, with and without the 10 kDa ultrafiltration device (UD10kDa). SEA_3_ was spiked at 10 ng/mL in milk, n = 3.

Food matrices, especially dairy products such as milk and cheese, contain high amounts of proteins and small molecules that may negatively impact the recovery of the immunoaffinity extraction. Ultrafiltration was reported as an effective method for the extraction of SEA [29], as it helps to remove small proteins, peptides and small molecules while also concentrating the toxin. We selected a 10 kDa cut-off ultrafiltration device, which was used before the immunopurification step. The first experiment in milk revealed a loss of the SEA toxin during ultrafiltration with a recovery of only about 50%, probably due to the non-specific binding of the toxin to the filter. Polyvinylpyrrolidone (PVP) or casein solutions were used to saturate the sites of adsorption to the filter, resulting in a complete SEA recovery with the undiluted commercial casein solution (Casein 10× from Thermofisher Scientific, Waltham, MA, USA) and a four-fold increase in signal intensity (Figure 2). 

**Figure 3:**
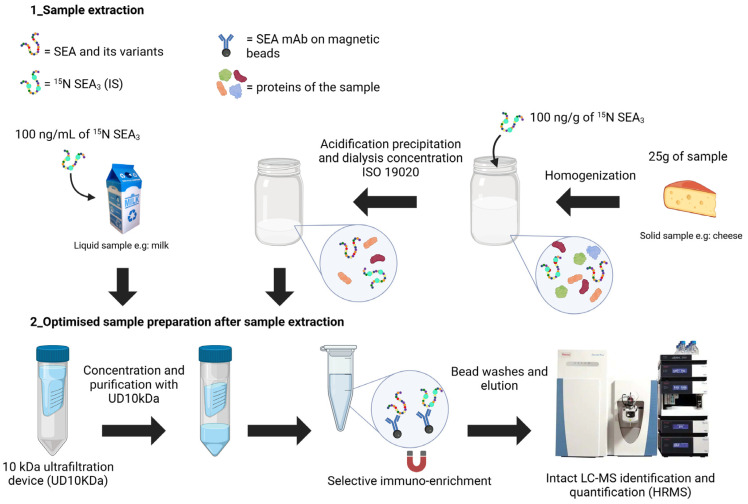
Sample preparation protocol for SEA followed by top-down analysis. Created in BioRender. https://BioRender.com/q37i192 accessed on 29 October 2024.

The immunoprecipitation (IP) step is based on magnetic beads functionalized with two antibodies of distinct epitopes to efficiently bind the four main SEA variants [17]. The initial volume of beads used for IP was 10 µL containing 500 µg/mL of antibody. Higher volumes, up to 40 µL, were tested to increase the capture efficiency of SEA spiked at 50 ng/mL in milk. A proportional increase of the signal was observed up to 20 µL of beads, then the signal remained constant (Figure S4). A volume of 20 µL of beads was, therefore, selected. The enhanced sample preparation protocol for milk, including ultrafiltration, immunoprecipitation and intact mass spectrometry, is indicated in Figure 3 (left side). The total extraction recovery of SEA during ultrafiltration and IP was determined at 50% (n = 3) by comparing the MS signal of the SEA_3_ variant spiked in milk before and after ultrafiltration and IP. 

**Figure 4:**
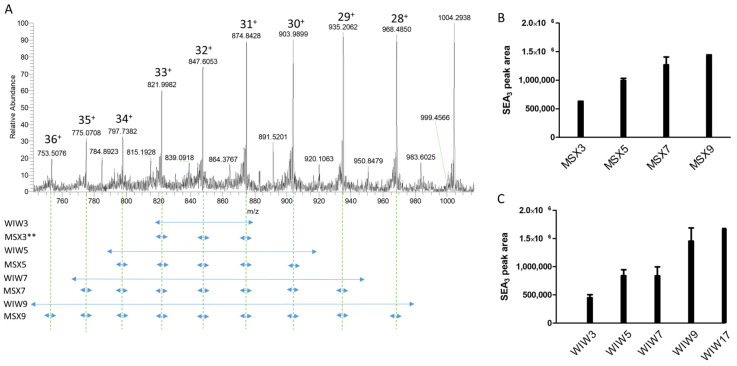
Isolation of multiple charge states for PRM acquisition. (**A**) MS spectra of native SEA_3_. (**B**) Peak area of SEA_3_ with 3 to 9 selected charge states using MSX. MSX was limited to 10 charge states with the Orbitrap instrument. (**C**) Peak area of SEA_3_ with 3 to 17 selected charge states using WIW isolation. In Supplementary Data Table S3, the different charge states isolated in MSX and WIW are reported. ** MSX3 isolation was previously used [17] and corresponded to the initial conditions in this work.

Mass spectrometry acquisition was also improved. The top-down detection method uses two distinct acquisition modes: a non-targeted full-scan mode and a targeted PRM mode. The full-scan mode enables the differentiation of SEA variants by determining the accurate masses of the intact proteins. The PRM mode allows a precise and specific measurement of the SEA concentration following MS/MS fragmentation of the protein into fragment ions. In this mode, an extracted ion chromatogram (XIC) signal of five selected fragment ions (Table S1) is generated. We have previously reported LODs at 5 ng/mL for the full-scan mode and 2.5 ng/mL for the PRM mode in simple matrices, buffer or strain supernatants [17]. The sensitivity of intact mass spectrometry is impacted by the signal dispersion resulting from the distribution of charge states (Figure 4A). To enhance the sensitivity of the PRM acquisition, we aimed to isolate multiple charge states of SEA in the quadrupole in front of the Orbitrap, followed by their simultaneous fragmentation in the higher-energy collisional dissociation (HCD) cell, to produce more intense fragment ions. In this objective, acquisition methods were developed to isolate in the quadrupole several charge states from native and ^15^N SEA_3_, either simultaneously by a single wide isolation window (WIW, from 10 to 700 Da wide) or sequentially by multiplex isolation of narrow windows of 8 Da (MSX, isolation of 3 to 9 charge states) (Table S2) [30]. We favored the isolation of charge states in the lower mass range, between z = 28 and z = 36, because of higher ion transmission efficiency by the quadrupole [31] and also considering that the signal of these charge states tended to increase at lower concentrations. In the first experiment, we confirmed that charge states z = 28, z = 32 and z = 36 of SEA_3_ produced the same fragment ions with similar relative abundances (Figure S5). Accumulated signals were then obtained either by the WIW or MSX isolations of between 3 and 9 charge states. As shown in Figure 4B, the signal intensity increased with the number of isolated charge states, reaching a maximum of nine charge states, which provided a threefold higher signal compared to the isolation of only three charge states by WIW or MSX. To further enhance the signal, we tested a broader selection window of 700 Da in the WIW isolation, corresponding to the isolation of nearly all of the detected charge states, from z = 36 to z = 19 (WIW17). The signal was not higher than the WIW of nine charge states (Figure 4B). When comparing MSX and WIW isolations for the selected nine charge states, we observed similar peak areas (Figure 4B). However, WIW allowed for the simultaneous isolation of the nine charge states from the ^15^N-labeled SEA_3_ and also all the reported SEA variants, ensuring their proper detection (Figure S6 and Table S3). We, therefore, selected the WIW isolation of nine charge states. In this condition, the elution peak was well defined with around 25 data points at 5 ng/mL (Figure S7).

### 2.3. Evaluation of the Final Protocol in Dairy Food Matrices

The final protocol combined all optimizations for sample preparation and mass spectrometry acquisition. We evaluated the protocol first in a protein-rich liquid matrix, e.g., milk, processed according to Figure 3 (left side). Milk was spiked with the SEA_3_ variant at concentrations ranging from 0.1 to 25 ng/mL to assess the limit of detection (LOD), dynamic range and repeatability. The ^15^N SEA_3_ toxin was spiked before the ultrafiltration step in all experiments.

**Figure 5:**
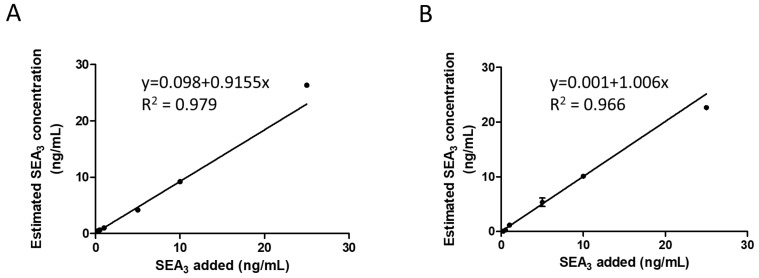
Linearity of the top-down assay for SEA_3_ spiked in milk and analyzed in PRM mode using the WIW9 isolation (**A**) or full-scan mode (**B**) (n = 4).

The LOD in full-scan mode was defined as the lowest concentration where the intact mass of the sequence variant could be determined, while in PRM mode, it corresponded to the lowest concentration where the signal exceeded three times the noise with detection of at least three of the five selected fragment ions. The method achieved an LOD at 0.5 ng/mL in PRM and 1 ng/mL in full-scan mode with a linear response up to 25 ng/mL (Figure 5), consistent with the concentrations found in contaminated products [18,19,20]. The repeatability of the PRM mode for SEA quantification was evaluated using the ratio of the peak area of SEA_3_ divided by the peak area of the labeled ^15^N SEA_3_. The coefficient of variation of the normalized peak area measured in six replicates of quality controls (QCs) spiked in milk at 1 and 5 ng/mL was below 13%, demonstrating good repeatability of the top-down protocol, even at a concentration near the LOD (Table S4). A measurement based on the peak area of SEA_3_ only, i.e., excluding the normalization by ^15^N SEA_3_, yielded a coefficient of variation of 22%. The ^15^N SEA_3_ toxin thus improved the assay precision by effectively mimicking the native toxin and undergoing the same extraction and MS analysis. 

The LOD at 0.5 ng/mL in milk represents a fivefold increase in sensitivity compared to our previous work [17]. While this improvement is significant, the final gain is somewhat lower than anticipated during the optimization of the individual protocol steps, probably due to the complexity of the matrix. Meanwhile, the top-down method has a comparable sensitivity to the bottom-up method [15], avoiding the time-consuming digestion by trypsin and allowing precise quantification. 

**Figure 6:**
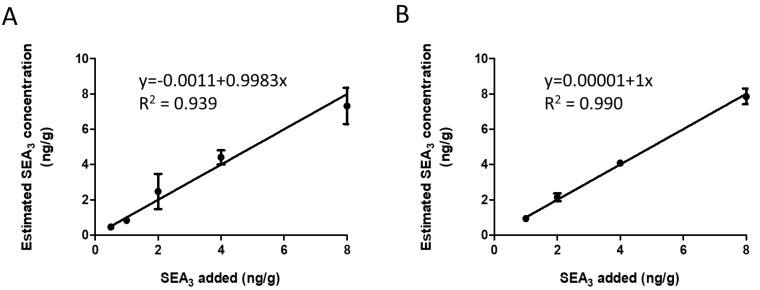
Linearity of SEA_3_ in Roquefort analyzed in PRM mode using the WIW9 isolation (**A**) or full-scan mode (**B**) (n = 2).

Evaluation was also performed in solid matrices, especially in diverse French cheeses. Linearity and LOD were established with the French cheese “Roquefort” analyzed either blank or spiked with concentrations ranging between 0.5 and 8 ng/g. To homogenize the solid cheese samples, the Standard EN ISO 19020 sample extraction protocol [32] was applied, which included acid precipitation and dialysis of 25 g of cheese, followed by our optimized ultrafiltration and immunocapture procedure as described in Figure 3 (right side). Full-scan and PRM modes were used for identification of the sequence variant and precise quantification of SEA level, respectively. The LOD in Roquefort was determined at 0.5 ng/g for PRM and 1 ng/g for full-scan, and linearity is illustrated in Figure 6A,B. 

A diversity of solid dairy products, including ice cream and four French cheeses, was used to evaluate further the top-down method. Each sample was received from ANSES (Maison-Alfort, France), either non-contaminated or artificially contaminated, at 8 ng/g with SEA_3_. We performed blind MS analysis to identify contaminated and non-contaminated samples, quantity of the toxin level and identity of the variant involved. The panel of products illustrates the diversity of dairy matrices, ranging from sweet (ice cream) and salty (Roquefort) to fatty (Roquefort, Emmental, Rocamadour), soft (Mozzarella) and hard (Emmental) textures. Positive and non-contaminated samples were identified by the method (Table 1). For quantification, concentrations were calculated using the external calibration curve established in Roquefort cheese, assuming similar matrix effects. The recovery rates, expressed as percentages in Table 1, exceeded 80% for three of the tested products, indicating a reliable estimate of the toxin concentration in these matrices. For Emmental and Mozzarella, the recovery rates were around 60%, suggesting some toxin loss during homogenization. In all samples, the identified variant by full-scan was SEA_3_, which matched with the spiked variant. The mass differences between the measured and theoretical masses, up to 3 Da, were attributed to the complexity of the matrix and the low level of contamination. 

The results demonstrated that the optimized top-down assay is suitable for the quantification of SEA in contaminated dairy products despite the challenges due to the variety of compositions and textures of these complex matrices. 

## 3. Discussion

The main objective was to improve the top-down assay reported by Lefebvre et al. (2021) [17] for the detection and quantification of SEA in dairy matrices usually involved in Staphylococcal food poisoning outbreaks, such as cheese and milk. Staphylococcal food poisoning outbreaks are a major cause of foodborne illness in Europe, and their notifications have been mandatory since 2005. Criteria for the enumeration of coagulase-positive Staphylococci (CPS) and the detection of staphylococcal enterotoxins (SEs) in cheese have been set down in Commission Regulation (EC) No.2073/2005 amended by Commission Regulation (EC) No.2019/229. The confirmed presence of SEs in any foodstuff represents a potential hazard to human health, as mentioned in Article 14 of Regulation (EC) No.178/2002.

Any contaminated batch is considered unsuitable for consumption and must be destroyed. Thus, contamination by SEs can have profound economic and media implications. For instance, in the United States, approximately 9.4 million foodborne illnesses are reported annually, with 1.3 million (14%) attributed to contamination by *Bacillus cereus*, *Clostridium perfringens* or *Staphylococcus aureus*. The economic burden of these cases is estimated at nearly USD 523 million per year [33,34]. Also, identifying the source of contamination promptly is crucial to prevent further outbreaks. Consequently, rapid and robust methods for detecting contaminated dairy food and tracing the source of contamination are essential.

Dairy samples constitute a real challenge for intact mass spectrometry because of the matrix complexity, containing highly abundant proteins or other molecules, a diversity of textures and the low amount of SEA usually detected in contaminated food linked to intoxication with levels reported at 0.5 ng/mL [19], 2.12 ng/g [20] or 3.7 ng/g [21]. For these reasons, we produced a stable isotope-labeled ^15^N SEA, spiked early in the protocol and mimicking the toxin to reach precise quantification. Protocol optimizations for higher sensitivity were performed on sample preparation and MS acquisition. The optimized workflow reached a detection limit as low as 0.5 ng/mL by PRM or 1 ng/mL by full-scan modes, showing a five-fold increased sensitivity compared to our previous work [17]. We showed that such trace amounts of the SEA toxin can be detected and quantified in complex dairy samples by the optimized protocol, making it applicable to food involved in SFPO episodes. An LOD at or below 1 ng/mL is a significant achievement for a top-down method, given the numerous challenges involved in the intact analysis of medium-size proteins. These challenges include signal dispersion due to the multiple charge states generated by electrospray ionization [35], protein loss during the liquid chromatography separation or low fragmentation efficiency [36]. Only a few top-down methods were reported at LOD below 1 ng/mL [37], and none were applied to SE toxins. As a consequence, bottom-up proteomics is generally more sensitive and typically constitutes the method of choice for biotoxin analysis by mass spectrometry [38,39,40,41]. Interestingly, the LOD of our improved top-down method is now similar to the most efficient bottom-up assays for SEs [12,42,43], while it has several advantages over bottom-up. The targeted PRM mode provides a specific and precise quantification of the toxin, avoiding the time-consuming trypsin digestion into peptides, thereby providing fast results within only about three hours. Furthermore, the non-targeted full-scan mode differentiates sequence variants without the need for prior isolation of *S. aureus* strains [17] and could potentially be used to monitor other post-translational modifications in the future [44,45]. The present top-down assay is the only proteomic method reported to date that can both detect the SEA variants and provide precise quantification of the toxin level in food samples. In addition, the risk of a false negative result due to an unexpected sequence variant is very unlikely for three reasons. First, we used a panel of two antibodies with distinct epitopes, previously shown to capture the four main SEA variants [17]. Second, the non-targeted full-scan mode on the high-resolution instrument can detect any modified amino acid in the sequence by principle. Finally, a wide isolation window of 250 Da, termed WIW9 isolation, spanning all known variants m/z, was selected for the targeted PRM acquisition. 

The improved top-down method could complement measurements by immuno-enzymatic assays [11,12] for confirmation of the presence of SEA, identification of the variant involved, and determination of the toxin level. Nevertheless, sensitivity is still in favor of reference ELISA measurements used for SFPO investigation, which achieves an LOD around 0.05 ng/mL for SEA [46] or even below 10 pg/mL [12]. Higher LC-MS sensitivity could be obtained in the future with nano-chromatography but at the cost of longer runtimes [47]. 

Before being adopted by food laboratories, the top-down assay requires full validation, including assessment of repeatability and reproducibility across several days and laboratories, determination of the limit of quantification, evaluation of matrix effects and uncertainty measurement. Then, the approach could also be extended to other SEs and implemented in reference food safety laboratories for application in real cases of food intoxication. For now, the assay is already suitable for research purposes and epidemiological studies of SEA variants, and also in situations of surprising false negative results by immunoassay or bottom-up MS. It could also be evaluated on other complex food samples such as mixed products containing meat, pizza or potted meat also described to be sources of SFPOs [48,49]. 

As a summary, in this study, we optimized top-down mass spectrometry conditions to achieve limits of detection (LOD) at or below 1 ng/mL. Additionally, we produced and incorporated a fully 15N-labeled staphylococcal enterotoxin A (SEA) standard early in the protocol, enabling accurate and reliable quantification of SEA. Finally, we demonstrated the applicability of the method for rapid and robust mass spectrometry identification of SEA in contaminated dairy products despite the challenges due to the variety of compositions and textures of these complex matrices. Of note, the top-down method offers the advantage of directly identifying and quantifying the toxin independently of any isolation or characterization of the *S. aureus* bacteria, as is necessary with genomic assays. 

## 4. Conclusions

Through optimizations to the sample preparation and signal acquisition protocols, we achieved detection limits comparable to those of traditional bottom-up mass spectrometry methods. Our findings confirm the top-down method’s reliability and effectiveness for identifying and quantifying low levels of SEA variants in complex dairy matrices.

## 5. Materials and Methods 

### 5.1. Chemicals and Reagents

Phosphate-buffered saline (PBS), phosphate-buffered saline Tween 10×, sodium phosphate monobasic and dibasic, phosphate monobasic and dibasic dodecahydrate, potassium phosphate, ammonium bicarbonate, polyéthylène glycol (PEG), polyvinylpyrrolidone (PVP), sodium acetate, sodium chloride, bovine serum albumin, dithiothreitol, iodoacetamide, 15N-labeled ammonium chloride, agar, glucose, M9 salts, magnesium sulfate, calcium chloride, isopropyl β-D-1-thiogalactopyranoside (IPTG), ampicillin, ultrafiltration devices Amicon^®^ ultra-4 (4 mL capacity, 10 kDa molecular cut-off), analytical grade formic acid, hydrochloric acid, sodium hydroxide, disodium hydrogen phosphate, sodium chloride and acetonitrile were from Sigma Chemical Co. (St. Louis, MO, USA). RapiGest SF was from Waters Corporation (Milford, MA, USA). Ultrapure water was obtained using a Milli-Q Plus purifier (Millipore, Bedford, MA, USA). Dynabeads M-280 tosylactivated magnetic beads and blocker casein in PBS were from Thermo Fisher Scientific (Waltham, MA, USA). Sequencing grade modified trypsin was from Promega (Fitchburg, WI). Murine monoclonal antibodies (mAb) mAb_SEA5 and mAb_SEA7 were produced in-house (CEA-LERI), as reported previously [12]. The native SEA_3_ toxin was obtained from Toxin Technology (Sarasota, FL, USA) and previously characterized [17].

### 5.2. Safety Precaution 

The experiments were conducted with strict compliance with safety protocols for handling toxic substances, including the use of appropriate personal protective equipment to ensure safety. SEA-contaminated solutions and consumables were deactivated by overnight treatment with 2 M NaOH. 

### 5.3. SEA Sequence Information 

The sequence of the SEA standard obtained from Toxin technology corresponds to the reference P0A0L2 (SEA_3_) in Uniprot (http://www.uniprot.org), accessed on 29 October 2024.

### 5.4. Production of the ^15^N Labeled SEA_3_

To obtain SEA_3_ protein labeled with ^15^N stable isotope, the *E. coli* bacteria commercially obtained from Invitrogen, Thermo Fisher Scientific, Illkirch, France was grown in an M9 minimal medium, with ^15^NH_4_Cl as the sole nitrogen source as described by Lippens [50] and 50 µg/mL ampicillin. 

Optimal ^15^N SEA_3_ production in the cytosolic fraction of *E. coli* reached 30 °C after induction with 100 μM IPTG for 22 h. The entire protocol of purification was described previously [12]. 

Protein purity was assessed by gel electrophoresis (Agilent protein 230 kit with Agilent 2100 Bioanalyzer, Agilent Technologies Inc., Santa Clara, CA, USA) and protein concentration was determined by measuring absorbance at 280 nm using a theoretical molecular extinction coefficient of 37,945 L·mol^−1^·cm^−1^ calculated with the sequence in Expasy. Mass spectrometry analyses were performed in intact mode and after trypsin digestion to characterize the labeled standard and confirm its mass and sequence. 

### 5.5. Food Samples

Calibration samples were prepared in milk and Roquefort cheese. In milk, the toxin SEA_3_ was spiked at 0, 0.1, 0.25, 0.5, 1, 2.5, 5 and 10 ng/mL to establish a calibration range and determine the method’s limit of detection (LOD). Quality control (QC) samples were analyzed in five replicates at 1 and 5 ng/mL. A calibration curve was also generated in Roquefort cheese, ranging from 0.5 ng/g to 8 ng/g. 

Five types of dairy products, including vanilla ice cream and four cheeses—Roquefort, Rocamandour, Mozzarella and Emmental—were contaminated at ANSES with 8 ng/g of SEA_3_. These matrices were then extracted following the EN ISO 19020 [32]. For signal normalization, 100 ng/g of ^15^N SEA_3_ was added to the food matrices at the beginning of the protocol (Figure 3). 

### 5.6. SE Extraction from Solid Food Matrices

The standard EN ISO 19020 protocol for food contaminated with Staphylococcus species was used to extract enterotoxins, as described in Nia et al. 2021 [32]. 

A 25 ± 0.1 g portion of the food matrix was weighed. For cheese and ice cream, the sample was ground and mixed with 40 mL of water, and the mixture was then acidified with HCl to achieve a pH of 3.5 to 4.0. After centrifugation at 3130× *g* for 15 min at 4 °C, the supernatant was neutralized to a pH of 7.4 to 7.6 using sodium hydroxide. A second centrifugation at 3130× *g* for 15 min at 4 °C was performed, and the resulting supernatant was dialyzed overnight (6–8 kDa) in a 30% (*w*/*v*) PEG solution. The protein retained in the dialysis membrane was recovered using 5 mL of PBS. The extract was then collected and placed in an ultrafiltration device with a 10 kDa cut-off (UD10 kDa). 

### 5.7. Ultrafiltration, Preparation of mAb-Coated Beads and Immuno-Affinity Extraction of SEA

Milk and standard EN ISO 19020 extracts from homogenized solid food matrices were collected in an ultrafiltration device with a cut-off at 10 kDa (UD10 kDa). The ultrafiltration device was previously saturated overnight (12 h) with a solution of blocker casein in PBS to limit toxin adhesion to tube walls. After the overnight saturation, the tube was centrifuged for 20 min at 4000× *g* at 4 °C to eliminate the casein solution. A volume of 4 mL of sample prepared following ISO 19020 protocol was then loaded and centrifuged for 20 min at 4000× *g* at 4 °C. The concentrated solution (1 mL) was collected for immuno-affinity purification. 

Two types of mAbs were used for this study, SEA5 and SEA7 [12]. They were coupled separately to Dynabeads M-280 tosylactivated magnetic beads using 16 μL (30 mg/mL) of beads for 4 μg of antibody and incubated overnight in PBS. To perform immunocapture, 20 µL of the mix of functionalized beads was added to 900 µL of extracted and ultracentrifuged food sample and 100 μL of PBS-Tween 10×. After incubation for 1 h at room temperature, successive bead washings were performed following the protocol described by [17]. 

### 5.8. Liquid Chromatography−High-Resolution Mass Spectrometry for Top-Down Quantification

An LC-MS system composed of an Ultimate 3000 chromatography system coupled to a Q-Exactive (Quadrupole-Orbitrap) mass spectrometer (Thermo Fisher Scientific, Courtaboeuf, France) was used for this study. An ACQUITY UPLC Protein BEH C4 reverse-phase column (300 Å, 1.7 μm, 2.1 mm × 150 mm, Waters Corporation Milford) was implemented for protein separation with a 15 min gradient at a constant temperature of 50 °C. Mobile phases and chromatographic gradient were the same as described in [17]. Eluted SEA toxin was introduced into the Q-Exactive instrument via an electrospray ionization source with a capillary voltage and temperature set to 4 kV and 320 °C, respectively. The instrument operated sequentially in targeted parallel reaction monitoring (PRM) followed by full-scan high-resolution mass spectrometry (HRMS) in positive-ion mode. For both acquisitions, ten microliters were injected into the LC-MS system. In the full-scan HRMS mode, the AGC target was set at 1 × 10^6^ ions, fill time at 170 ms and Orbitrap resolution at 17,500 at 200 *m*/*z*. The PRM mode targeted the nine most abundant charge states using the WIW9 mode, as reported in Table S2 in Supplementary Data. The AGC target was set at 1 × 10^6^, fill time at 512 ms and Orbitrap resolution at 140,000 at *m*/*z* 200 (fwhm) with an isolation window at 250 *m*/*z*. Collision energy in the HCD cell was set at 18%. 

### 5.9. Data Analysis

The area ratio of SEA with the internal standard (^15^N SEA_3_) was determined. Xcalibur 2.2 (Thermo Fisher Scientific, Waltham, MA, USA) was used to reprocess the full-scan data and based on the area ratio of the extracted-ion chromatogram (XIC) of the nine most intense charge states (28, 29, 30, 31, 32, 33, 34, 35, 36) using a mass extraction window of 0.2 *m*/*z*. The *m*/*z* values used are reported in Table S3 in Supplementary Data. 

The XIC signals of up to the selected five fragments (b11^2+^, b18^4+^, b19^4+^, b20^4+^, b21^4+^) obtained in PRM mode were summed to increase the MS signal (Figure S3). Quantification relied on external calibration curves prepared in milk and Roquefort cheese, spiked with increasing concentrations of the SEA_3_ standard and constant concentration of the internal standard (^15^N SEA_3_). 

## Figures and Tables

**Table 1:** Top-down analysis of five types of dairy products non-contaminated or contaminated with 8 ng/g of SEA_3_. French cheeses and ice cream were prepared according to the protocol for solid matrices in Figure 3. The interpolated concentration represents the recovered amount, determined by plotting the measured PRM signal on the calibration curve established for Roquefort (Figure 6). The variant was identified by the full-scan mode [17].

	SEA_3_ Added (ng/g)	Interpolated Concentration (ng/g)	Recovery (%)	Measured Mass (Da)	Mass Difference (Da) *	Identified Variant
Roquefort_1	8.00	6.87	86	27,090.37	2.15	SEA_3_
Roquefort_2	0	<LOD	NA	NA	NA	NA
Rocamandour_1	8.00	6.66	83	27,090.00	2.52	SEA_3_
Rocamandour_2	0	<LOD	NA	NA	NA	NA
Vanilla ice cream_1	8.00	7.28	91	27,090.79	1.73	SEA_3_
Vanilla ice cream_2	0	<LOD	NA	NA	NA	NA
Emmental_1	8.00	5.32	66	27,089.69	2.83	SEA_3_
Emmental_2	0	<LOD	NA	NA	NA	NA
Mozzarella_1	8.00	4.94	62	27,092.46	0.06	SEA_3_
Mozzarella_2	0	<LOD	NA	NA	NA	NA

* Theoretical mass (MH+) of SEA3: 27,092.52 Da, <LOD: Below the limit of detection, NA: Not applicable.

## Data Availability

The original contributions presented in the study are included in the article/Supplementary Materials; further inquiries can be directed to the corresponding author.

## Supplementary Materials

The following supporting information can be downloaded athttps://www.mdpi.com/article/10.3390/toxins16120535/s1, Figure S1: Optimization of recombinant ^15^N SEA_3_ protein production conditions; Figure S2: Electrophoresis analysis (1D-SDS-PAGE) of different fractions of ^15^N SEA_3_ after purification; Figure S3: LC separation and MS spectra of the three best-responding peptides for SEA_3_ and ^15^N SEA_3_, Figure S4: Optimization of the volume of beads for immunoprecipitation. Samples were prepared in milk spiked with 50 ng/mL of SEA_3_ (n = 4 for each condition); Figure S5: MS^2^ spectra of charge states z = 28, z = 32, z = 36 of intact SEA_3_; Figure S6: MS/MS spectra of intact SEA_3_ and ^15^N SEA_3_ after WIW9 isolation at RT 5.6 min; Figure S7: Chromatogram of SEA_3_ showing data points across the LC elution peak; Table S1: Fragment ions identified in the HRMS/MS spectra of SEA_3_ and ^15^N SEA_3_; Table S2: Q-Extractive parameters tested for acquisition in the PRM mode; Table S3: Theoretical average masses and m/z ratios of the nine charge states (z = 28 to z = 36) for the eight SEA variants; Table S4: Coefficients of variation of SEA_3_ peak area and SEA_3_/^15^N SEA_3_ ratio [17,51,52].
