# Supplementary material for: Quantification of Staphylococcal Enterotoxin A Variants at Low Level in Dairy Products by High-Resolution Top-Down Mass Spectrometry
Source: Toxins (Basel). 2024 Dec 11;16(12):535. doi: 10.3390/toxins16120535 (PMC11679111; doi:10.3390/toxins16120535)
Supplement: Supplementary file 1 [file toxins-16-00535-s001.zip › toxins-3315482-supplementary.pdf]

Supplementary Materials: Quantification of Staphylococcal Enterotoxin A Variants at Low Level in Dairy Products by High-Resolution Top-Down Mass Spectrometry

Nina Aveilla, Cécile Feraudet-Tarisse, Dominique Marcé, Abdelhak Fatihi, François Fenaille, Jacques-Antoine Hennekinne, Stéphanie Simon, Yacine Nia and François Becher

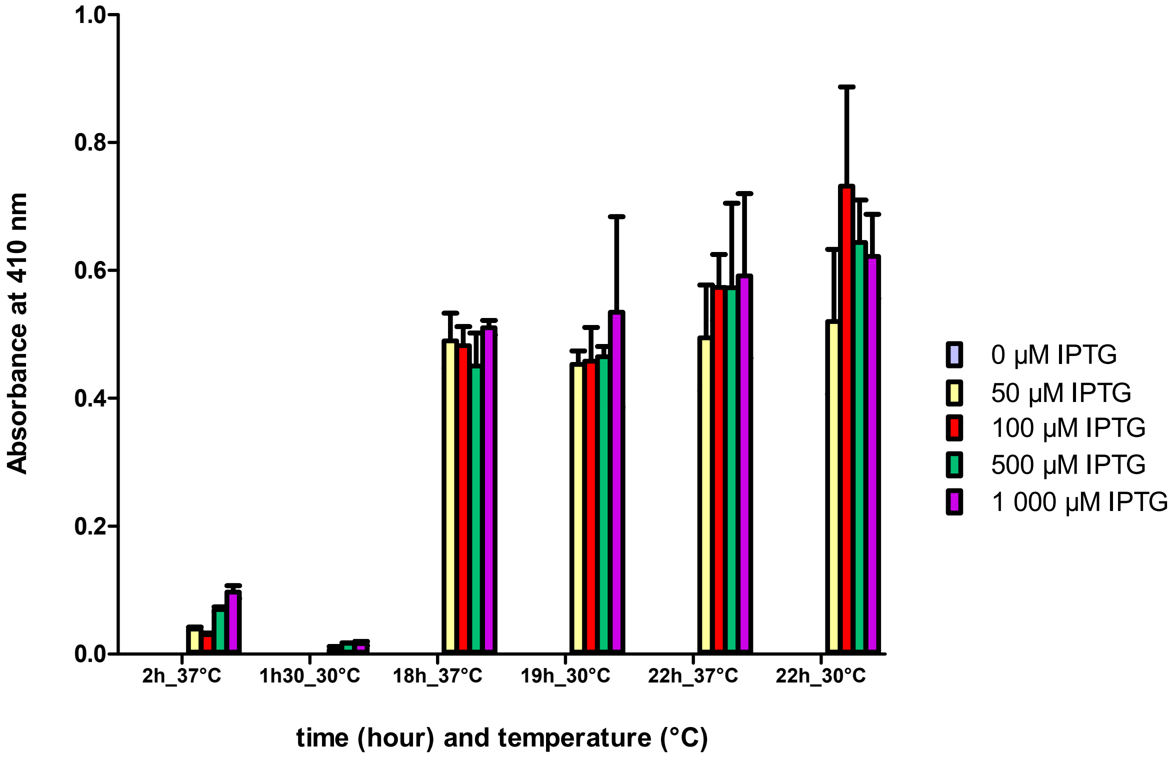

Figure S1. Optimization of recombinant ^{15}N SEA_3 protein production conditions.

A direct enzyme-linked immunosorbent assay (ELISA) [1] method was employed to estimate the relative protein level expression in culture supernatants. The absorbance signal at 410 nm increased proportionally with the concentration of SEA. Expression of transformed *E. coli* BL21 (DE3) pLysS bacteria was performed at two temperatures: 30 °C and 37 °C in M9 minimal medium with $^{15}NH_4Cl$ as the nitrogen source and 50 µg/mL ampicillin. Five concentrations of IPTG were tested for induction. During the pilot culture kinetics, 1 mL samples were taken at 1 h 30, 2 h, 18 h, 19 h and 22 h and centrifuged at 2500 g for 20 min. Cytosol proteins were extracted from bacterial pellets and were analyzed by direct ELISA to compare relative ^{15}N SEA_3 expression levels between the different culture conditions.

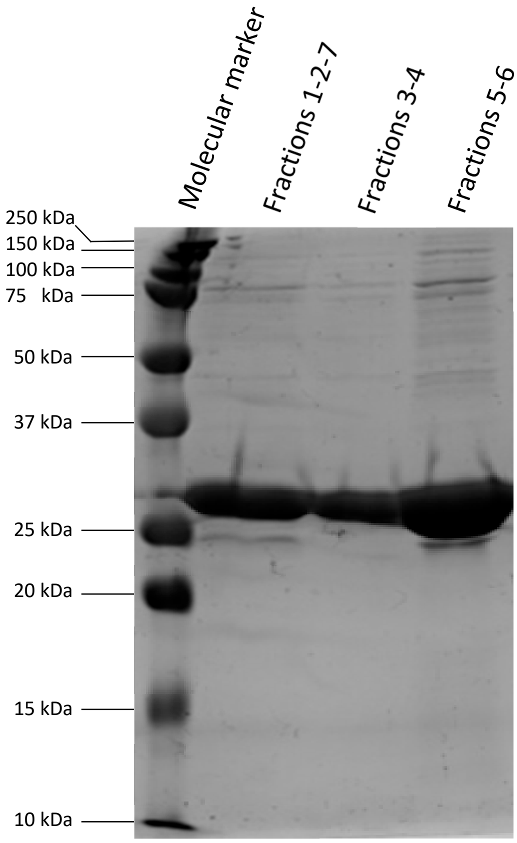

Figure S2. Electrophoresis analysis (1D-SDS-PAGE) of different fractions of ^{15}N SEA$_3$ after purification.

The mass of our labeled protein is expected to be around 28 kDa. A one-liter culture of the labeled SEA was purified thanks to its poly-histidine tag by nickel affinity chromatography [2].

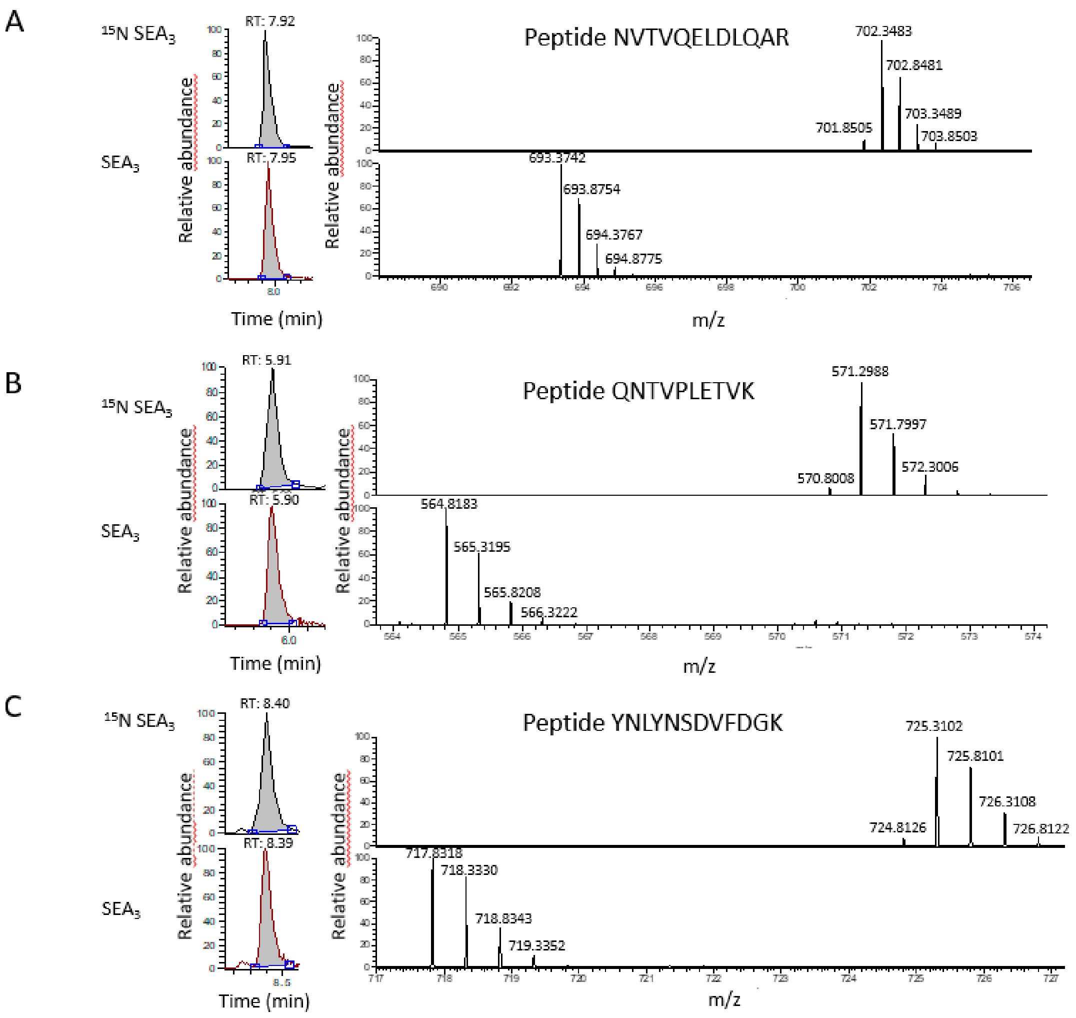

Figure S3. LC separation and MS spectra of the three best-responding peptides for SEA_3 and ^{15}N SEA_3.

The mass shift between labeled and unlabeled corresponds to ≈100% labeling efficiency. Electrophoresis analysis (1D-SDS-PAGE) of different fractions of ^{15}N SEA_3 after purification.

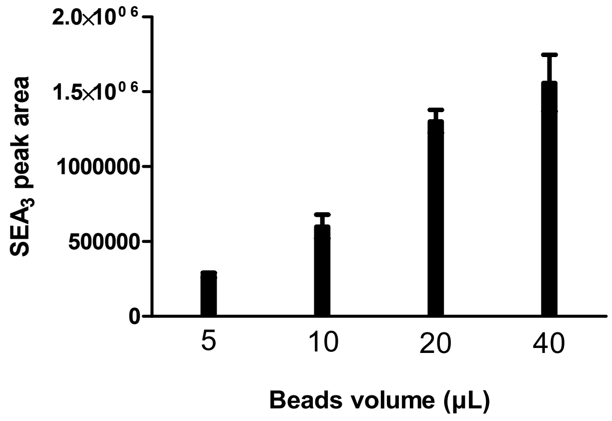

Figure S4. Optimization of the volume of beads for immunoprecipitation. Samples were prepared in milk spiked with 50 ng/mL of SEA_3 (n = 4 for each condition)

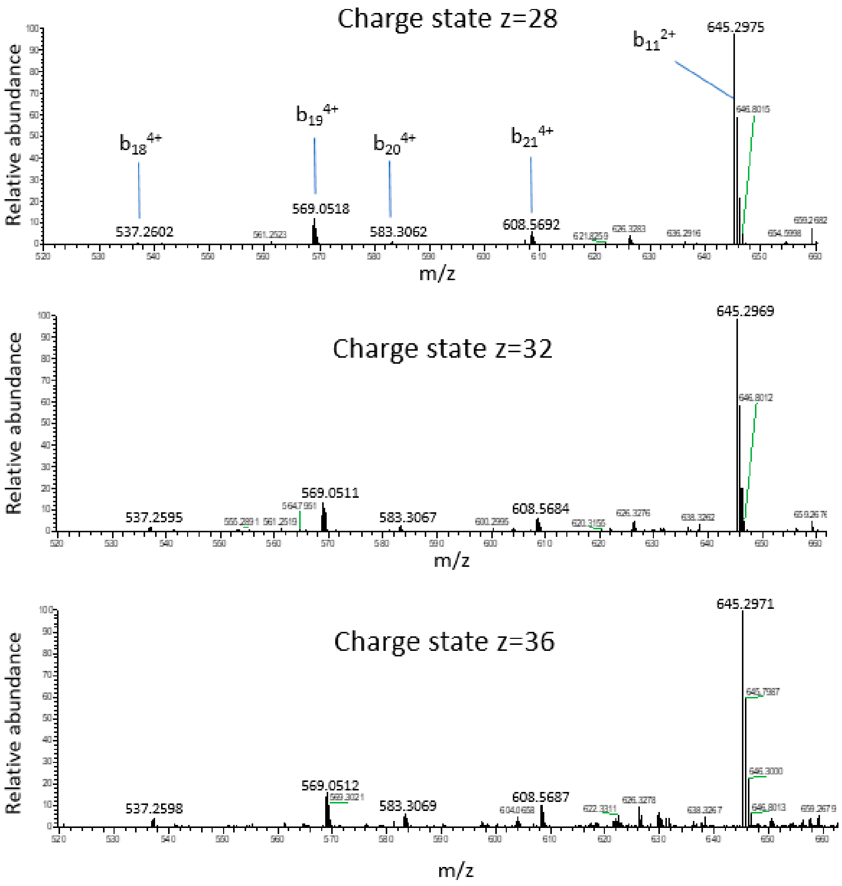

Figure S5. MS[2] spectra of charge states z=28, z=32, z=36 of intact SEA3.

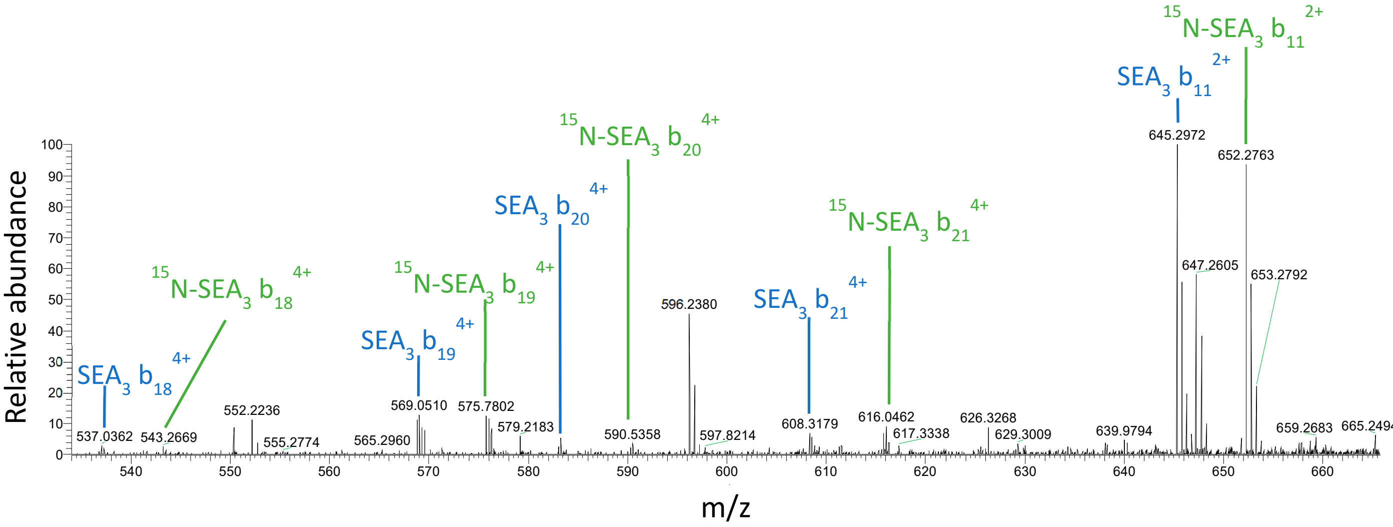

Figure S6. MS/MS spectra of intact SEA3 and ^{15}N SEA3 after WIW9 isolation at RT 5.6 min.

The simultaneous isolation of SEA3 and ^{15}N SEA3 with the WIW9 mode resulted in pairs of labeled/unlabeled fragment ions in the MS/MS spectra.

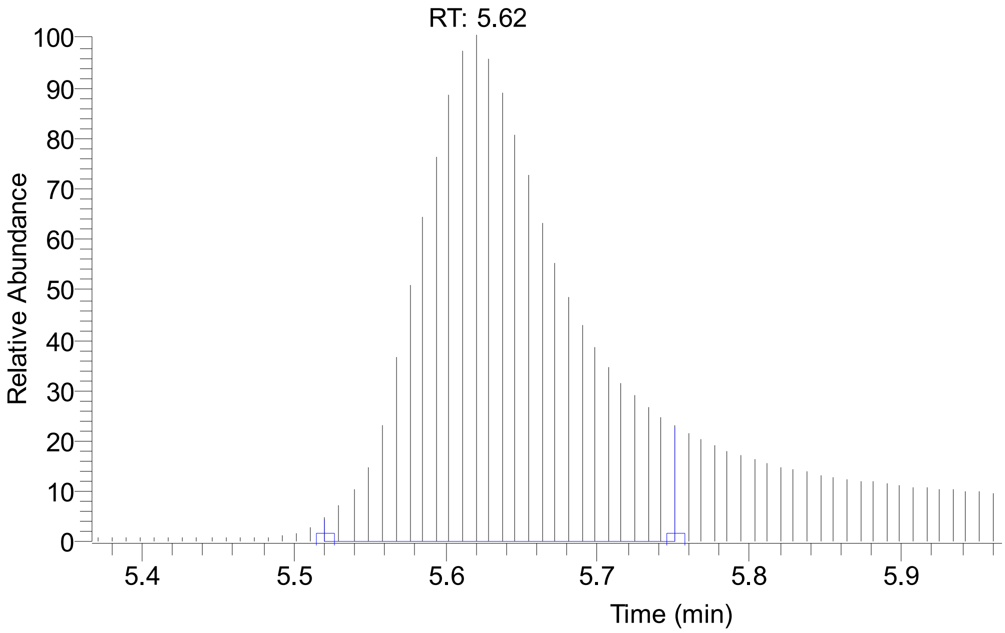

Figure S7. Chromatogram of SEA_3 showing data points across the LC elution peak.

Table S1. Fragment ions identified in the HRMS/MS spectra of SEA_3 and ^{15}N SEA_3.

			SEA_3 m/z		^{15}N SEA_3 m/z	
	**Fragment ion sequence**	Chemical formula	Experimental	Theoretical	Experimental	Theoretical
b_{11}^{2+}	SEKSEEINEKD	$C_{52}H_{87}N_{14}O_{25}$	645.2974	645.2964	652.2783	652.2788
b_{18}^{4+}	SEKSEEINEKDLRKKSEL	$C_{90}H_{157}N_{26}O_{35}$	536.7867	536.7853	543.2682	543.2660
b_{19}^{4+}	SEKSEEINEKDLRKKSELQ	$C_{95}H_{165}N_{28}O_{37}$	568.8011	568.7999	575.7813	575.7792
b_{20}^{4+}	SEKSEEINEKDLRKKSELQG	$C_{97}H_{168}N_{29}O_{38}$	583.0566	583.0553	590.2868	590.2838
b_{21}^{4+}	SEKSEEINEKDLRKKSELQGT	$C_{101}H_{175}N_{30}O_{40}$	608.3180	608.3172	615.7970	615.7950

Comparison of experimental and theoretical monoisotopic m/z. The theoretical m/z was obtained by MS-product (https://prospector.ucsf.edu/prospector/cgi-bin/msform.cgi?form=msproduct).

Table S2. Q-Extractive parameters tested for acquisition in the PRM mode.

Method	Isolated charge states (z)	Orbitrap resolution	Fill time (ms)	Isolation window (Da)	MSX	Duty cycle (ms)
MSX3*	30, 31, 32	140 000	170*3	8	3	512
MSX5	29, 30, 31, 32, 33	140 000	102*5	8	5	512
MSX7	28, 29,30, 31, 32, 33, 34	140 000	73*7	8	7	512
MSX9	28, 29, 30, 31, 32, 33, 34, 35, 36	140 000	56*9	8	9	512
WIW3	30, 31, 32	140 000	512	60	/	512
WIW5	29, 30, 31, 32, 33	140 000	512	130	/	512
WIW7	28, 29,30, 31, 32, 33, 34	140 000	512	186	/	512
WIW9	28, 29, 30, 31, 32, 33, 34, 35, 36	140 000	512	250	/	512
WIW17	From 19 to 36	140 000	512	700	/	512

MSX refers to multiplex mode, where specific charge states are isolated within distinct mass windows. WiW stands for wide isolation window mode, where several charge states of SEA are isolated in a single wide mass window.

*: MSX3 isolation was used previously [3] and corresponded to the initial condition in this work.

Table S3. Theoretical average masses and m/z ratios of the nine charge states (z=28 to z=36) for the eight SEA variants.

Theoretical m/z (Da) of all reported SEA variants at z=28 to z=36*										
	**MH^{+1} (av)**	**MH^{+28}(av)**	**MH^{+29}(av)**	**MH^{+30}(av)**	**MH^{+31}(av)**	**MH^{+32}(av)**	**MH^{+33}(av)**	**MH^{+34}(av)**	MH^{+35}(av)	MH^{+36}(av)
SEA$_1$	27012.443	965.7730	932.5052	901.4553	872.4086	845.1773	819.5964	795.5202	772.8199	751.3806
SEA$_2$	26936.347	963.0553	929.8812	898.9187	869.9539	842.7993	817.2904	793.2821	770.6457	749.2669
SEA$_3$	27092.527	968.6331	935.2667	904.1247	874.9919	847.6799	822.0232	797.8756	775.1080	753.6052
SEA$_4$	27042.470	966.8454	933.5406	902.4562	873.3772	846.1156	820.5063	796.4034	773.6778	752.2147
SEA$_5$	26135.622	934.4579	902.2700	872.2279	844.1240	817.7766	793.0260	769.7314	747.7678	727.0245
SEA$_6$	27005.361	965.5201	932.2610	901.2192	872.1801	844.9560	819.3818	795.3119	772.6175	751.1839
SEA$_7$	27092.527	968.6331	935.2667	904.1247	874.9919	847.6799	822.0232	797.8756	775.1080	753.6052
SEA$_8$	26980.357	964.6270	931.3988	900.3857	871.3735	844.1746	818.6241	794.5765	771.9031	750.4894

Quadripole selection range with the WIW9 mode is from *m/z* 722.60 to 972.60 Da.

* Calculated using MS product

Table S4. Coefficients of variation of SEA$_3$ peak area and SEA$_3$/^{15}N SEA$_3$ ratio.

	Coefficient of variation (%)	
**SEA concentration**	Without ^{15}N SEA$_3$	With ^{15}N SEA$_3$
1 ng/mL	22%	13%
5 ng/mL	7%	4%

Six quality controls of SEA in milk at two levels of concentration, 1 and 5 ng/mL, were analyzed by PRM.

References

1. Costopoulou, D.; Leondiadis, L.; Czarnecki, J.; Ferderigos, N.; Ithakissios, D.S.; Livaniou, E.; Evangelatos, G.P. Direct ELISA Method for the Specific Determination of Prothymosin Alpha in Human Specimens. *J. Immunoassay Immunochem.* **1998**, doi:10.1080/01971529808005487.
2. Spriestersbach, A.; Kubicek, J.; Schäfer, F.; Block, H.; Maertens, B. Chapter One - Purification of His-Tagged Proteins. In *Methods in Enzymology*; Lorsch, J.R., Ed.; Laboratory Methods in Enzymology: Protein Part D; Academic Press, 2015; Vol. 559, pp. 1–15.
3. Lefebvre, D.; Fenaille, F.; Merda, D.; Blanco-Valle, K.; Feraudet-Tarisse, C.; Simon, S.; Hennekinne, J.-A.; Nia, Y.; Becher, F. Top-Down Mass Spectrometry for Trace Level Quantification of Staphylococcal Enterotoxin A Variants. *J. Proteome Res.* **2022**, *21*, 547–556, doi:10.1021/acs.jproteome.1c00886.
